# PARP inhibitor-induced anti-tumour chemokine response is suppressed by dipeptidyl peptidase 4 (DPP4) in ovarian cancer

**DOI:** 10.1038/s41416-025-03076-4

**Published:** 2025-06-27

**Authors:** Christoph Stange, Tobias F. Dreyer, Maximilian Riedel, Franziska Elsen, Stefanie Seitz, Dorine Hamann, Marion Kiechle, Holger Bronger

**Affiliations:** 1 Department of Gynecology and Obstetrics, Technical University of Munich, Munich, Germany; 2 German Cancer Consortium (DKTK), partner site Munich, a partnership between DKFZ and Technical University of Munich, Munich, Germany

**Keywords:** Ovarian cancer, Cancer therapeutic resistance, Targeted therapies

## Abstract

**Background:**

Inhibitors of poly(ADP-ribose) polymerase (PARPi, e.g. olaparib) induce a tumour-suppressive chemokine release *via* STING in homologous recombination deficient (HRD) and proficient (HRP) cancers.

**Methods:**

Dose-dependent effects of olaparib on HRD (ID8-*Brca2*^*(−/−)*^) and HRP (ID8) ovarian cancer cell proliferation and chemokine release. Survival of immunocompetent and immunocompromised ID8 mouse models treated with different olaparib doses. Inhibition and overexpression of the chemokine-inactivating dipeptidyl peptidase 4 (*mDPP4*) in HRD and HRP mouse models. Correlation of hDPP4 immunohistochemistry staining with survival in 208 high-grade serous ovarian cancer patients.

**Results:**

In our study, olaparib induced the chemokines mCCL5 and mCXCL10 in a dose-dependent manner in HRD and HRP ovarian cancer cells. An optimised olaparib concentration induced chemokine release and improved survival in the syngeneic HRD ovarian cancer mouse model but not in immunocompromised mice, likely promoting synergism of immune activation and tumour cell cytotoxicity. Overexpression of mCCL5- and mCXCL10-cleaving *mDPP4* induced resistance to olaparib in the HRD mouse model. Conversely, mDPP4 inhibition led to the reversal of intrinsic PARPi resistance in the HRP mouse model.

**Conclusions:**

This study highlights the immune system-activating properties of PARP inhibitors and suggests harnessing these for effective PARPi therapy in ovarian cancer, especially in the context of HRP disease.

## Background

In recent years, inhibitors of the poly(ADP-ribose) polymerase (PARPi) have revolutionised the treatment of advanced ovarian cancer [1]. Unprecedented improvements in survival have been achieved, particularly in patients with homologous recombination deficient (HRD) tumours, e.g., mediated by BRCA1/2 loss-of-function mutations. This has been explained primarily by so-called synthetic lethality, that is, cell death following the dual inactivation of BRCA1/2 and PARP1 and a trapped PARP1 lesion at the replication fork [2]. Accordingly, in the PAOLA-1 study, maintenance therapy with the PARP inhibitor olaparib following first-line chemotherapy led to a significant survival benefit only in patients with HRD tumours [3]. However, the PRIMA trial, testing niraparib in a similar setting, also demonstrated therapy response in homologous recombination proficient (HRP) tumours, which underpins earlier positive results in HRP recurrent ovarian cancer [4, 5]. The PARPi effect in HRP tumours challenges synthetic lethality as the sole mechanism of action, and the authors of the PRIMA trial already suspected that other mechanisms, such as immune activation, might explain their results.

A full understanding of the PARPi mode of action on a molecular level is needed to extend the use of PARPi to HRP patients. This represents an urgent clinical need, as 50% of all advanced ovarian cancers are HRP [6].

Much evidence now supports that PARPi can modulate the immune system in *BRCA*-deficient and wild-type models [7–9]. Activation of the cGAS/STING pathway by PARPi-induced accumulation of double-stranded DNA fragments in the cytosol of tumour cells initiates a type I interferon-like response with the release of the T cell-recruiting chemokines CCL5 and CXCL10, but also the upregulation of PD-L1 [10–12]. Chemokine-driven immune activation could explain the efficacy of PARP inhibitors in HRP ovarian cancer. Furthermore, immune activation offers a rationale for the synergism of PARPi and immune checkpoint inhibition observed in ovarian cancer clinical trials [13–15]. Preclinical evidence demonstrates the dependence of successful immune checkpoint inhibition on chemokines such as those released upon STING activation [16, 17].

The present work aimed to further elucidate the importance of STING activation for PARPi efficacy in ovarian cancer. To this end, we used chemokine release as a robust surrogate for STING activation in vivo. Our studies suggest that STING-mediated immune activation might contribute to PARPi efficacy, even in HRD ovarian cancer. In support of this, we show that proteases such as dipeptidyl peptidase 4 (DPP4), which is capable of cleaving STING-induced chemokines, mediate resistance to PARP inhibitors. Conversely, PARPi resistance in HRP ovarian cancer can be overcome by DPP4 inhibition.

## Materials and methods

### Human tissue samples and patient characteristics

Fresh-frozen and formalin-fixed, paraffin-embedded tumour samples from 208 patients with high-grade serous ovarian cancer (HGSOC) (FIGO III and IV), treated at the Department of Gynecology and Obstetrics (Technical University of Munich, Munich, Germany) between 1990 and 2014, were included. The study was approved by the Institutional Review Board of the Technical University of Munich (Munich, Germany; approval 392/17S) in accordance with the Declaration of Helsinki. Written informed consent was obtained from all patients. None of the patients received neoadjuvant chemotherapy before debulking surgery. Patient characteristics are given in Table S1. Formalin-fixed, paraffin-embedded tumour specimens were mounted on slides to generate tissue microarrays for immunohistochemical studies, as described previously [18]. Fresh-frozen tumour samples (*n* = 33) were mechanically dissected and lysed for RNA or protein extraction. When the extracted proteins were to be used for DPP4 activity assays, no protease inhibitors were used in the protein extraction process.

### Immunohistochemistry of human tumour tissue

DPP4 immunohistochemistry was performed using tissue microarrays (TMAs) with triplicates of formalin-fixed, paraffin-embedded (FFPE) tumour sections from each patient. The tissue was sliced into 3 µm sections and mounted on slides. The slides were deparaffinised by xylene and hydrolysed by successive incubations in aqueous solutions with a descending alcohol content (100–50%). Heat-induced antigen retrieval was performed in citrate buffer (pH 6.0) before endogenous peroxidase activity was quenched with 3% (v/v) H_2_O_2_ (20 minutes). This was followed by blocking unspecific antibody binding sites with 5% goat serum in antibody diluent (ZUC025-500, Zytomed).

Samples were then incubated with anti-human DPP4 rabbit IgG (NB100-59021, Novus) (1:150 in antibody diluent solution) for 1 hour at room temperature. For detection, the polymer one-step system (Zytomed) and 3,30-diaminobenzidine (DAB, Zytomed) as substrate were used. All slides were stained with hematoxylin (Merck) and washed with TBST (0.1% Tween 20) between each step. We evaluated the results by using a semiquantitative score based on the staining intensity of the tumour tissue: absent (0), weak (1), moderate (2), or strong (3). Evaluators were blinded to the clinical data. Histological images were taken using the digital slide scanner NanoZoomer Digital Pathology RS (Hamamatsu, Japan) and semiquantitatively analysed with NDP.view 2 software version 2.8.24.

### Immunohistochemistry of mouse tumour tissue

Mouse tissue was fixed in 4% paraformaldehyde for 48 hours at room temperature and stored in phosphate-buffered saline (PBS) at 4 °C before paraffin embedding. Granzyme B staining was performed by the Comparative Experimental Pathology core facility (School of Medicine, TUM) on an automated immunostainer (Agilent Technologies) as described previously [17], using citrate buffer at pH 6.0 for heat-induced epitope retrieval and a dilution of 1:1000 for the polyclonal rabbit granzyme B antibody (ab4059, Abcam). QuPath open-source software version 0.4.3 [19] was used to evaluate DAB-positive cells by adapting the workflow from Paik et al. [20]. Briefly, all slides of the same animal experiment were preprocessed by adjusting stain vectors and selecting the whole tumour area on each slide. Then, the chosen area was divided into tiles of 300 micrometres, and positive cell detection was run using the parameters optical density sum, 200 µm^2^ maximum area, 1 µm cell expansion, and the score compartment nucleus: DAB OD mean. The threshold was adapted for each slide individually to values between 0.15 and 0.28. All tiles with less than 200 detected cells were discarded, and the average number of positive cells was taken from the five tiles with the highest percentage of positive cells per slide, classified as regions of interest (roi).

### Cell lines

ID8 wild-type cells, ID8-*Trp53*^*(−/−)*^ and ID8-*Trp53*^*(−/−)*^*Brca2*^*(−/−)*^ were kindly provided by Prof. Iain McNeish’s laboratory (University of Glasgow) [21] and cultured in high glucose Dulbecco's Modified Eagle's Medium (DMEM, 61965059, gibco) supplemented with 4% (v/v) foetal bovine serum (FBS, 10270106, gibco), 1% (v/v) N-2-hydroxyethylpiperazine-N-2-ethane sulphonic acid (HEPES, 15630056, gibco), and 1% (v/v) ITS-G (41400045, gibco). All cell lines were maintained until passage number 20 in a cell incubator in a humidified 5% CO_2_ atmosphere at 37 °C, confluence was maintained below 100%. Mycoplasma contamination was monitored regularly using MycoStrip (rep-mys-50, InvivoGen).

### MTT assay

2500 cells per well were seeded in triplicates for each condition into 96 well plates (83.3924, Sarstedt) in fully supplemented culture media. For dose-dependent experiments, the media was exchanged the next day with a specific volume of fully supplemented culture media and supplemented with the indicated reagents, followed by incubation in the cell incubator. After 0 h, 24 h, 48 h, and 72 h, MTT (3-(4,5-dimethylthiazol-2-yl)-2,5-diphenyltetrazolium bromide, 0.83 mg/ml final concentration, M2128-1G, Sigma) was added to each well of one of the 96 well plates and incubated for 2.5 h in the cell incubator. 150 µl DMSO (D2650, Sigma-Aldrich) was added to each well, followed by 10 min incubation in the cell incubator. Absorbance was measured after 5 min of plate shaking at a wavelength of 570 nm using a Multiskan FC (Thermo Fisher Scientific, USA). Results were normalised to DMSO controls. Means of three biological replicates were compared. Significant differences were determined by two-way ANOVA with Tukey’s multiple comparisons test.

### PARPi-mediated stimulation of mCCL5 and mCXCL10

3 x 10^4^ cells were seeded into 12 well plates in fully supplemented culture media. The next day, cells were stimulated with parp inhibitors olaparib (S1060, SelleckChem), niraparib (S2741, SelleckChem), talazoparib (S7048, SelleckChem), rucaparib camsylate salt (kindly provided by Clovis) and the sting inhibitor H151 (0.5 µg/ml final concentration, inh-h151, InvivoGen) in DMEM supplemented with 5% (v/v) FBS. After 72 h of incubation in the cell incubator, cell supernatant was collected for ELISA analysis.

### ELISA to assess chemokine concentrations

To assess chemokine concentrations in cell supernatants or ascites samples, mCCL5/RANTES (DY478, R&D), mCXCL9 (DY492, R&D), and mCXCL10 (DY466, R&D) Duoset ELISAs were used according to the manufacturer's instructions. Samples were handled on ice before the start of the assay to limit protein degradation.

### Generation of *DPP4*-overexpressing and empty vector cell lines

*DPP4* overexpression was introduced into ID8 cell line derivatives using the ViraSafeTM Lentiviral Expression System (Neo) (VPK-213-ECO, Cell Biolabs). The mouse *DPP4* construct BC022183 was excised from a *DPP4*-containing vector (*DPP4* (BC022183) Mouse Untagged Clone, MC200935, OriGene) and ligated into the transfer vector pSMPUW-Neo. *DPP4*-pSMPUW-Neo or pSMPUW-Neo-empty vector control were transfected together with kit plasmids pRSV-Rev, pCMV-Eco and pCgpV into the packaging cell line HEK293T and incubated for 6 h in the cell incubator before changing media. 24 h later, virion-containing supernatant was collected, filtered, and used to infect ID8 cell lines. After selecting single-cell clones resistant to 1 mg/ml G418 (11811-031, Thermo Fisher), qPCR and DPP4 activity assays were performed to detect single-cell clones positive for *DPP4* integration. Additionally, MTT assays and ELISA were performed to assess olaparib-induced chemokine concentrations. We selected *DPP4*-overexpressing and empty vector control cells that behaved similarly in these assays. We also selected one ID8-*Trp53*^*(−/−)*^*Brca2*^*(−/−)*^ empty vector control clone with similar MTT assay-assessed proliferation as the parental ID8-*Trp53*^*(−/−)*^*Brca2*^*(−/−)*^ cells, but less sensitivity to olaparib-induced secretion of mCCL5 and mCXCL10, which we named ID8-*Trp53*^*(−/−)*^*Brca2*^(−/−)^*.

### Mouse experiments

Eight- to 10-week-old wild-type female C57BL/6JRj were obtained from Janvier, and eight- to 10-week-old female athymic nude mice from Charles River Laboratory. Mice were maintained in a pathogen-free animal facility, and all animal procedures and experiments were approved by the Government of Upper Bavaria (Regierung von Oberbayern) and followed the institutional guidelines of the Technical University of Munich (Munich, Germany).

1 x 10^7^ ID8 cell line derivatives, solved in Dulbecco's Balanced Salt Solution (DPBS, 14190-094, gibco), were injected intraperitoneally. Mice were then randomly selected and numbered by earmarks to avoid differences in administered tumour cells due to the duration of the cell injection procedure.

Mice receiving sitagliptin treatment were treated 5x per week with sitagliptin (sitagliptin phosphate monohydrate, 1757, BioVision) in DPBS by oral gavage. Sitagliptin concentration was adjusted to 50 mg/kgBW. 1 mg sitagliptin was administered as a 50 µl solution with a 20 mg/ml concentration. Mice receiving olaparib treatment were treated 5x weekly with 50 µl olaparib (SelleckChem) *i.p*. in a solution containing DMSO, PEG300 (807484, Sigma-Aldrich), and H_2_O in the corresponding volume ratios 4/3/3.

Mice were sacrificed after reaching previously defined humane endpoint criteria. Ascitic fluid was collected by *i.p.* aspiration and centrifuged at 10.000 × *g* at 4 °C for 10 min. The supernatant was used for further analysis. Sizable primary tumours were generally found next to the ovaries, in the mesentery, and on the diaphragm and collected in embedding cassettes as formalin-fixed paraffin-embedded tissue for immunohistochemical analyses or as fresh frozen tissue for RNA or protein isolation. The sample size for each condition was 7 unless stated otherwise in the figure legend.

### DPP4 activity assay

DPP4 activity was analysed using the synthetic DPP4 substrate analogue H-glycyl-prolyl-AMC (Gly-Pro-AMC, #40.025.200.025, Bachem) as described previously [22]. Briefly, in Optiplate-96 black well plates (#6005270, PerkinElmer) on ice, 20 µl of assay buffer (100 mM NaCl, 100 mM Tris, pH 7.8) was mixed with 25 µl of sample protein lysate adjusted to a protein content of 30 µg. Then, 5 µl 40 µM 1G244 (#Y0432, AKSci) or a mixture of 2.5 µl 80 µM 1G244 and 2.5 µl 80 µM Sitagliptin (#1757, Absource) were added. To start the reaction, 50 µl 200 µM substrate Gly-Pro-AMC was added, and fluorescence was determined using a spectrofluorometer (VictorX, Tecan) with an excitation wavelength of 355 nm and an emission wavelength of 460 nm over one hour at room temperature. DPP4 activity is presented as relative fluorescent units per minute (RFU), which was calculated by subtracting the RFU of samples mixed with sitagliptin and 1G244 (inhibition of DPP4, DPP8, and DPP9) from the RFU of samples mixed with 1G244 (inhibition of DPP8 and DPP9) and divided by the duration of the measurement in minutes.

### Quantification and statistical analysis

Statistical analyses were performed using GraphPad Prism Software version 9.0.1 (GraphPad Software). In vitro experiments were conducted with three technical replicates at least three times unless stated otherwise. Bars and horizontal lines represent the mean ± standard error of the mean (SEM). Specific statistical tests are indicated in the corresponding figure text. *P* values <0.05 were considered significant.

For retrospective *DPP4*/*BRCA2* survival analyses, we used the publicly available Affymetrix dataset (retrievable at www.kmplot.com [23]) using the best cutoff as selected by the programme.

## Results

### STING-mediated chemokine release exhibits differential sensitivity to olaparib in *Brca2-*deficient and non-deficient murine ovarian cancer cells

**Fig. 1:**
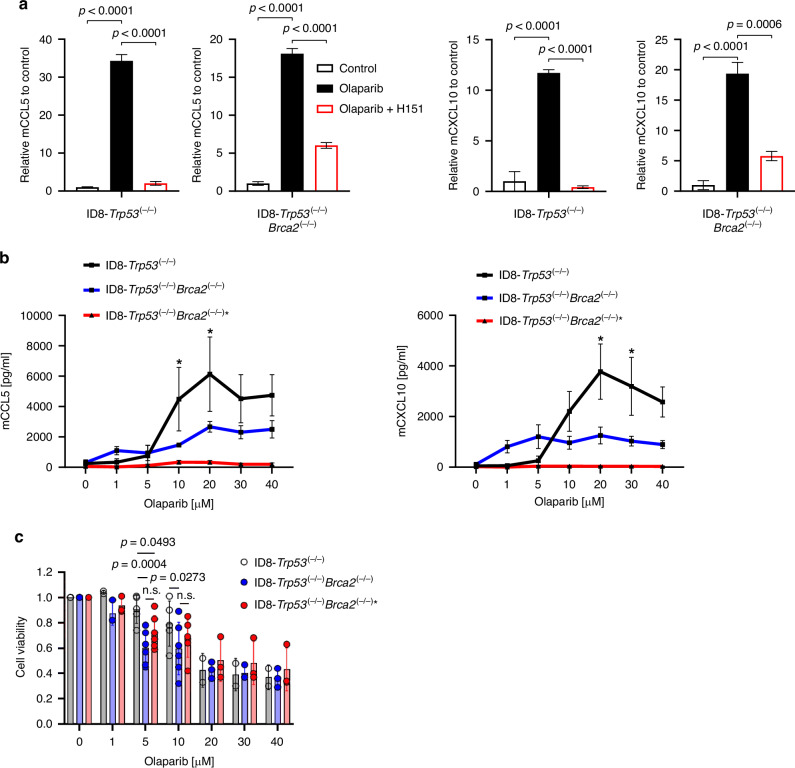
Olaparib induces chemokine release in ID8 ovarian cancer cell lines irrespective of Brca2-knockout. **a** Cell lines ID8-Trp53^(−/−)^ and ID8-Trp53^(−/−)^Brca2^(−/−)^ were stimulated with 10 µM olaparib with or without 0.5 µg/ml H151 for 72 h. Supernatants were collected and mCCL5 and mCXCL10 concentrations were analysed *via* ELISA. One representative of three individual experiments with three technical replicates each is shown, values are normalised to their respective control, significant differences were determined by one-way ANOVA with Tukey’s multiple comparisons test. **b** ELISA-quantification of supernatant mCCL5 and mCXCL10 after stimulation of ID8-Trp53^(−/−)^, ID8-Trp53^(−/−)^Brca2^(−/−)^ and ID8-Trp53^(−/−)^Brca2^(−/−)^* with the indicated olaparib concentrations for 72 h. Means of three individual experiments are shown, significant differences were determined between ID8-Trp53^(−/−)^ and ID8-Trp53^(−/−)^Brca2^(−/−)^ by two-way ANOVA with Fisher's LSD test. **c** Viability of indicated ID8 cell lines after 72 h of incubation with the indicated olaparib concentrations as measured *via* MTT assay, normalised to DMSO control, means of at least three individual experiments are shown, significant differences were determined by two-way ANOVA with Tukey’s multiple comparisons test. If not stated otherwise, all figure results represent the mean, error bars are SEM, alpha = 0.05, *p<0.05.

In recent years, PARP inhibitors have been described to mediate the release of the two T-cell recruiting chemokines CCL5 and CXCL10 by activating the STING pathway in cancer cells [8–11]. To further study these effects in ovarian cancer, we stimulated the two murine ovarian cancer cell lines ID8-*Trp53*^*(−/−)*^ and ID8-*Trp53*^*(−/−)*^*Brca2*^*(−/−)*^ with the PARP inhibitor olaparib (10 µM) for 72 h and measured chemokine concentrations in the cell supernatants. Olaparib elicited a significant induction of mCCL5 and mCXCL10 in both cell lines, which was significantly reduced by the STING inhibitor H151 (0.5 µg/ml) (Fig. 1a). Similar effects were observed when using the PARP inhibitors talazoparib (1 µM), niraparib (10 µM), or rucaparib (10 µM) (Fig. S1A). Although quantitative differences in chemokine induction between cell lines may be influenced by other factors (e.g. cytotoxicity, see below), olaparib-induced chemokine secretion in ID8 cell lines appears to be STING-dependent.

Next, we investigated chemokine release as a function of increasing olaparib concentrations after 72 h of treatment. Up to concentrations of 5 µM olaparib, the release of mCCL5 and mCXCL10 into the cell supernatant was markedly higher in ID8-*Trp53*^*(−/−)*^*Brca2*^*(−/−)*^ cells (Fig. 1b). However, at olaparib concentrations of 10 µM and above, mCCL5 and mCXCL10 concentrations in ID8-*Trp53*^*(−/−)*^ cell supernatants significantly surpassed those of ID8-*Trp53*^*(−/−)*^*Brca2*^*(−/−)*^ cells at the same cell seeding density, as a plateau was reached at lower olaparib concentrations in the ID8-*Trp53*^*(−/−)*^*Brca2*^*(−/−)*^ cells. The reason for reaching this plateau phase at lower olaparib concentrations may be the higher sensitivity of ID8-*Trp53*^*(−/−)*^*Brca2*^*(−/−)*^ to olaparib-induced cytotoxicity through synthetic lethality because of the BRCA2 knockout [24], leading to a loss of chemokine-producing cell mass, which was recapitulated by a significant reduction of cell viability upon 5 µM olaparib (*P*  =  0.0004) and 10 µM olaparib (*P*  =  0.0273) as compared to ID8-*Trp53*^*(−/−)*^ cells (Fig. 1c).

Of note, we compared concentrations of mCCL5 and mCXCL10 in supernatants from the parental ID8 wild-type cell line [25] with ID8-*Trp53*^*(−/−)*^ and ID8-*Trp53*^*(−/−)*^*Brca2*^*(−/−)*^. The chemokine concentrations in ID8 wild-type supernatants were about twice as high as in ID8-*Trp53*^*(−/−)*^ or ID8-*Trp53*^*(−/−)*^*Brca2*^*(−/−)*^ supernatants, at basal levels as well as when stimulated with different olaparib concentrations (Fig. S1B), consistent with previous reports describing activation of the STING signalling pathway by wild-type p53, for example through TREX1 degradation [26].

To better differentiate STING activation from the effect of synthetic lethality in further experiments, we screened for ID8-*Trp53*^*(−/−)*^*Brca2*^*(−/−)*^ cells that showed a strongly diminished chemokine induction upon PARP inhibition (hereinafter referred to as ID8-*Trp53*^*(−/−)*^*Brca2*^(−/−)^* cells, cf. red lines in Fig. 1b, c). This cell line secreted significantly less mCCL5 and mCXCL10 upon direct STING activation by the STING agonist DMXAA (Fig. S1C), but demonstrated a similar response to olaparib-induced cell cytotoxicity as ID8-*Trp53*^*(−/−)*^*Brca2*^*(−/−)*^ cells (Fig. 1c). Being sensitive to olaparib-induced cell cytotoxicity but insensitive to the induction of mCCL5 and mCXCL10 by olaparib, we used it as a model to further characterise the role of olaparib-induced chemokine release in vivo.

### PARPi-induced chemokine release correlates with improved survival in the *Brca2-*deficient ovarian cancer model in vivo

**Fig. 2:**
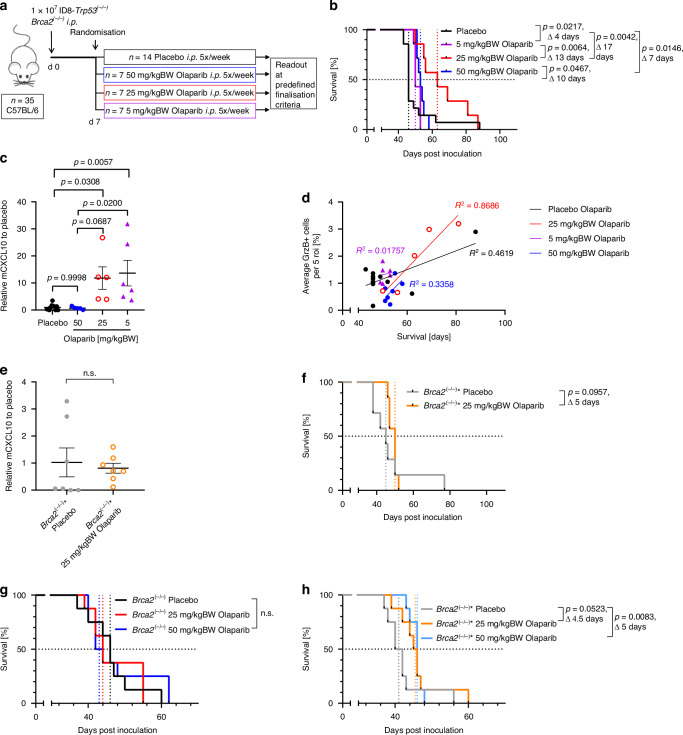
Olaparib induces mCXCL10 secretion in vivo. A high mCXCL10-inducing olaparib dose is beneficial for mouse survival. **a** Animal experiment design to investigate the effect of different olaparib doses, pooled from two individual experiments. **b** Kaplan-Meier plot showing survival of C57BL/6 mice intraperitoneally inoculated with syngeneic ID8-Trp53^(−/−)^Brca2^(−/−)^ and treated according to (**a**). **c** ELISA-quantification of mCXCL10 in the ascitic fluid taken at the time of animal sacrifice from (**b**), pooled from two individual experiments and normalised to the corresponding mCXCL10 mean in the placebo group, significant differences were determined by one-way ANOVA with Tukey’s multiple comparisons test, two samples from the placebo group were excluded as outliers by Grubb’s test. **d** Correlation of immunohistochemistry staining of granzyme B in tumour slides with mouse survival from (**b**), colours of the dots match the treatment groups from (**b**), roi = regions of interest. **e** ELISA-quantification of mCXCL10 in the ascitic fluid taken at the time of animal sacrifice from C57BL/6 mice intraperitoneally inoculated with ID8-Trp53^(−/−)^Brca2^(−/−)^*, treated with 25 mg/kgBW olaparib (*n* = 7) or placebo (*n* = 7), pooled from two individual experiments and normalised to the corresponding mCXCL10 mean in the placebo group, significant differences were determined by unpaired t-test with Welch's correction. **f** Kaplan-Meier plot showing survival of mice from (**e**). **g** Kaplan-Meier plot showing survival of athymic nude mice intraperitoneally inoculated with ID8-Trp53^(−/−)^Brca2^(−/−)^ treated with 25 mg/kgBW olaparib (*n* = 8), or 50 mg/kgBW olaparib (*n* = 8), or placebo (*n* = 8). **h** Kaplan-Meier plot showing survival of athymic nude mice intraperitoneally inoculated with ID8-Trp53^(−/−)^Brca2^(−/−)^* treated with 25 mg/kgBW olaparib (*n* = 8), or 50 mg/kgBW olaparib (n=8), or placebo (*n* = 8). The statistical significance of Kaplan-Meier analyses was calculated using the Log-rank test or the Gehan-Breslow-Wilcoxon test when groups crossed each other. ELISA results represent the mean, error bars are SEM, with alpha = 0.05, n.s. = not significant.

Next, we studied the role of olaparib-induced chemokine release in ovarian cancer in vivo. To this end, immunocompetent C57BL/6 mice were inoculated intraperitoneally with ID8-*Trp53*^*(−/−)*^*Brca2*^*(−/−)*^ cells and treated with different doses of olaparib (5, 25, and 50 mg/kgBW) five times a week, starting at day seven after tumour implantation (Fig. 2a). Mice treated with 50 mg/kgBW olaparib, which is a commonly used dosage in the literature [27–29], showed a significant survival benefit of seven days (median survival 46 vs. 53 days, *P* =  0.0146; Fig. 2b). To analyse the immune-activating effect of this PARPi treatment, mCCL5 and mCXCL10 were measured in the ascites of tumour-bearing mice as a surrogate for tumour-derived chemokines in the ID8 model [17], and which have been shown to correlate with T cell infiltration [30, 31]. However, neither mCCL5 nor mCXCL10 was elevated in the ascites after treatment with 50 mg/kgBW olaparib (Figs. 2c, S2A). Based on our in vitro observations, we hypothesised that sufficient chemokine release might be suppressed due to extensive tumour cell lethality at this dosage. This made us explore whether altered olaparib concentrations induce these chemokines in vivo and improve the survival benefit by an additive effect of chemokine-mediated immune activation and cytotoxicity.

With 5 mg/kgBW olaparib, a survival benefit of 4 days (median survival 46 vs. 50 days, *P* =  0.0217; Fig. 2b) was observed. This was not significantly different from the survival of mice treated with 50 mg/kgBW. With a dose of 25 mg/kgBW olaparib, however, the survival benefit was extended significantly to 17 days (median survival 46 vs. 63 days, *P* =  0.0042; Fig. 2b). In contrast to the highest dose, 5 mg/kgBW and 25 mg/kgBW resulted in a significantly higher mCXCL10 ascites concentration than in placebo-treated animals (Fig. 2c), despite a comparable tumour burden at the end of the experiment. mCCL5 was not significantly induced by olaparib at any dose (Fig. S2A). Moreover, 25 mg/kgBW olaparib led to a higher number of granzyme B-positive immune effector cells, well correlating with survival (Fig. 2d).

To further decipher the role of chemokine induction and immune activation in the overall olaparib therapy effect, we inoculated immunocompetent C57BL/6 mice with ID8-*Trp53*^*(−/−)*^*Brca2*^(−/−)^* tumour cells susceptible to olaparib-induced cytotoxicity but not to chemokine induction. In line with our in vitro results, we did not observe an mCXCL10 or mCCL5 induction in the ascites of the tumour-bearing mice despite using an olaparib dose of 25 mg/mgBW (Figs. 2e, S2B). Moreover, the survival benefit of 17 days, which we had observed with this treatment in the ID8-*Trp53*^*(−/−)*^*Brca2*^*(−/−)*^ model, shrunk to 5 days (median survival 45 vs. 50 days, *P* =  0.0957; Fig. 2f). This is close to the survival benefit we saw in the ID8-*Trp53*^*(−/−)*^*Brca2*^*(−/−)*^ model with an olaparib dose of 50 mg/kgBW (Fig. 2b), so it seems to reflect the pure effect of PARPi-mediated cytotoxicity.

We then employed the immunocompromised athymic nude mouse model that lacks mature T cells. In this model and in strong contrast to the immunocompetent mouse model, treatment of ID8-*Trp53*^*(−/−)*^*Brca2*^*(−/−)*^ tumours with 25 or 50 mg/kgBW olaparib did not mediate a survival benefit (Fig. 2g). Interestingly, similarly treated ID8-*Trp53*^*(−/−)*^*Brca2*^(−/−)^* tumours did benefit from olaparib treatment (Fig. 2h), but the effect was at best as high as postulated for the sole effect of olaparib cytotoxicity. Thus, chemokine induction and the hereby triggered adaptive immune response might be one component of enhanced olaparib efficacy at 25 mg/kgBW.

### Dipeptidyl peptidase 4 (DPP4) confers resistance to olaparib in *Brca2*-deficient murine ovarian cancer

Dipeptidyl peptidase 4 is a protease that cleaves two peptides off the N-terminus of CCL5 and CXCL10, thereby decreasing recognition by their cognate receptors CCR1 and CCR3 or CXCR3, respectively [32]. CXCR3, in turn, is responsible for the recruitment of tumour-suppressive T-cells to the ovarian cancer tumour microenvironment [17, 18]. DPP4 inhibitors are readily available and FDA-approved [33]. Furthermore, DPP4-typical cleavage products of CXCL10 were detected in human ovarian carcinoma [34].

Since endogenous *mDPP4* expression is not detectable in ID8-*Trp53*^*(−/−)*^*Brca2*^*(−/−)*^ cells, we transduced *mDPP4* into this cell line and selected cell clones positive for DPP4 activity (Fig. S3A). The clones and their respective control clones exhibited a similar sensitivity to olaparib regarding mCCL5 and mCXCL10 induction and a comparable proliferation behaviour (Fig. S3B–D).

**Fig. 3:**
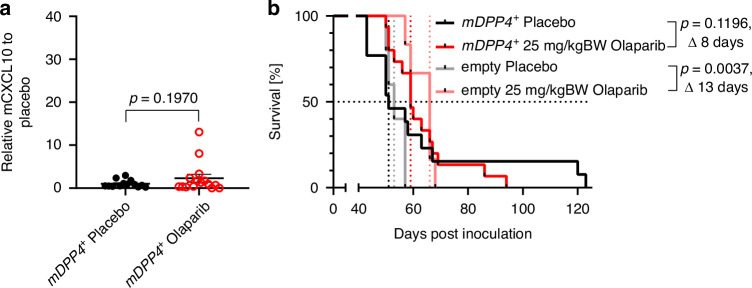
mDPP4 overexpression leads to olaparib resistance in HRD ID8 tumours. **a** ELISA-quantification of mCXCL10 in the ascitic fluid taken at the time of animal sacrifice from C57BL/6 mice inoculated with mDPP4-overexpressing ID8-Trp53^(−/−)^Brca2^(−/−)^mDPP4^+^ treated with 25 mg/kgBW olaparib (*n* = 15) or placebo (*n* = 13), pooled from two individual experiments and normalised to mCXCL10 mean in the corresponding placebo group. **b** Kaplan-Meier plot showing survival of C57BL/6 mice from 3(**a**) and, additionally, empty vector control cells treated with 25 mg/kgBW olaparib (*n* = 6) or placebo (*n* = 5). Statistical significance of Kaplan-Meier analyses was calculated using the Log-rank test or the Gehan-Breslow-Wilcoxon test when groups crossed each other. ELISA results represent the mean, error bars are SEM, significant differences were determined by unpaired t-test with Welch's correction.

Next, we injected the ID8-*Trp53*^*(−/−)*^*Brca2*^*(−/−)*^*mDPP4*^*+*^ cells into immunocompetent C57BL/6 mice and treated them with 25 mg/kgBW olaparib or placebo as described above. As seen in our models before, mCCL5 was not induced in the ascitic fluid (Fig. S3E). The striking induction of mCXCL10 upon olaparib in the ascites of mice bearing the ID8-*Trp53*^*(−/−)*^*Brca2*^*(−/−)*^ tumours (Fig. 2c) was almost completely lost in mice harbouring *mDPP4*-overexpressing tumours (Fig. 3a). This might be explained by the fact that mDPP4-cleaved mCXCL10 can act as a substrate for other proteases present in the heterogenous tumour environment in vivo which mediate general protein degradation [35], thereby escaping detection by our ELISA assay. *mDPP4*-overexpressing tumours became resistant to olaparib (25 mg/kgBW) as the significant survival benefit seen before (Fig. 2b) was largely reduced (median survival 51 vs. 59 days, *P* =  0.1196), while the survival benefit was retained in empty vector controls (median survival 53 vs. 66 days, *P* =  0.0037; Fig. 3b).

### mDPP4 inhibition with sitagliptin improves olaparib therapy in *Brca2* non-deficient murine ovarian cancer

**Fig. 4:**
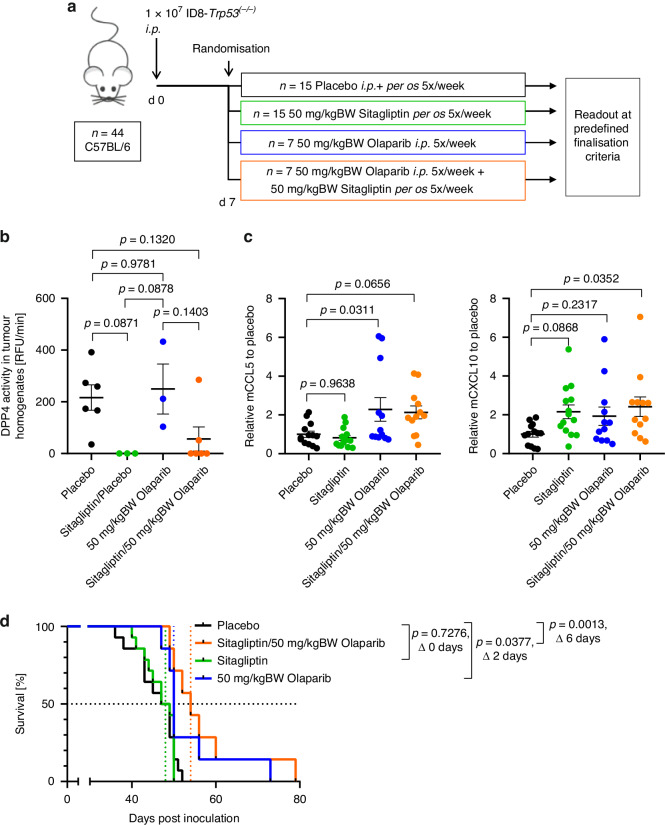
Combination treatment of olaparib with sitagliptin improves control of Brca2-wild-type tumours. **a** Animal experiment designed to investigate the effect of the combination treatment of olaparib with sitagliptin, placebo and sitagliptin groups pooled from two separate experiments. **b** DPP4 activity assay with tumour homogenates collected at the time of animal sacrifice from the animal experiment displayed in (**a**) without pooled placebo and sitagliptin groups, significant differences were determined by one-way ANOVA with Tukey’s multiple comparisons test. **c** ELISA-quantification of mCCL5 or mCXCL10, respectively, from the animal experiment displayed in (**a**), displayed relative to placebo, pooled from two individual experiments and normalised to the mCCL5 or mCXCL10 means in the corresponding placebo group, significant differences were determined by one-way ANOVA with correction for multiple comparisons (Dunnett). **d** Kaplan-Meier plot showing survival of C57BL/6 mice from the animal experiment displayed in (**a**). The statistical significance of Kaplan-Meier analyses was calculated using the Log-rank test or the Gehan-Breslow-Wilcoxon test when groups crossed each other. ELISA results represent the mean, error bars are SEM, n.s. = not significant, alpha = 0.05.

Since the induction of mCXCL10 could be part of the beneficial effect of olaparib in the *Brca2*^*(−/−)*^ model, and as we observed that *Brca2* wild-type ID8-*Trp53*^*(−/−)*^ cells could be stimulated by olaparib to induce this chemokine as well, we wondered whether olaparib efficacy could be improved in *Brca2* wild-type tumours by optimising olaparib dosage and adding a DPP4 inhibitor. Since we observed a mCXCL10 induction in ID8-*Trp53*^*(−/−)*^ at higher olaparib doses than in ID8-*Trp53*^*(−/−)*^*Brca2*^*(−/−)*^ (Fig. 1b), we used 50 mg/kgBW olaparib to treat C57BL/6 mice intraperitoneally inoculated with ID8-*Trp53*^*(−/−)*^ (Fig. 4a).

Adding sitagliptin to the four-armed experiment reduced DPP4 activity in the tumours (Fig. 4b), most likely by reducing the activity in non-tumour cells, as ID8-*Trp53*^*(−/−)*^ cells showed no DPP4 activity in vitro (Fig. S3F). In the ascites of mice treated with sitagliptin in combination with olaparib, both mCCL5 and mCXCL10 concentrations were increased (Fig. 4c), albeit with borderline statistical significance in the case of mCCL5. Of note, olaparib as a single therapy also significantly induced mCCL5 and moderately but not significantly, mCXCL10.

The survival of mice was only slightly enhanced with olaparib alone (median survival 48 vs. 50 days, *P* =  0.0377; Fig. 4d). Sitagliptin alone did not affect survival. However, the combination of both drugs led to a significant survival benefit (median survival 48 vs. 54 days, *P* =  0.0013; Fig. 4d), thus in a range comparable to what was seen with olaparib in the *Brca2*-deficient model (Fig. 2b).

### DPP4 is a feasible therapeutic target in human high-grade serous ovarian cancer

**Fig. 5:**
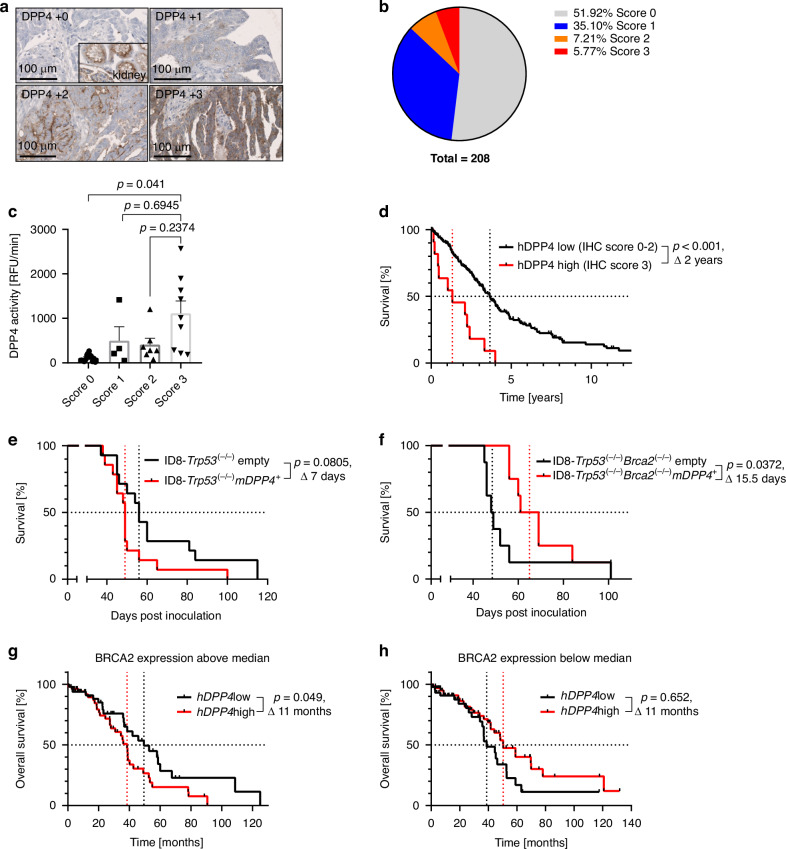
hDPP4 is overexpressed in HGSOC and correlates with a bad prognosis. In animal experiments and the TCGA dataset, this is dependent on the BRCA2-mutation status. **a** IHC hDPP4 staining was performed with tissue microarray samples from 208 patients with HGSOC. Depicted images were used as reference images to evaluate the hDPP4 staining. **b** Pie chart showing the distribution of staining intensities from (**a**). **c** Activity assay displaying DPP4 activity in fresh-frozen tumour samples from patients of the cohort from (**a**) (*n* = 33), categorised into the staining groups determined in (**b**) score 0 (*n* = 13), score 1 (*n* = 4), score 2 (*n* = 7), and score 3 (*n* = 9), three outliers were excluded by the ROUT method with Q = 1%. **d** Kaplan-Meier analysis of patient overall survival stratified into the hDPP4 low expressing group (score 0-2 from (**b**), *n* = 196) and the hDPP4 high expressing group (score 3 from (**b**), *n* = 12). **e** Kaplan-Meier plot showing survival of C57BL/6 mice intraperitoneally inoculated with syngeneic ID8-Trp53^(−/−)^mDPP4^+^ (*n* = 14) or ID8-Trp53^(−/−)^ empty (*n* = 14) pooled from three individual experiments. **f** Kaplan-Meier plot showing survival of C57BL/6 mice intraperitoneally inoculated with syngeneic ID8-Trp53^(−/−)^Brca2^(−/−)^mDPP4^+^ (*n* = 8) or ID8-Trp53^(−/−)^Brca2^(−/−)^ empty (*n* = 8). **g** KM-plotter [23] survival analysis with the following parameters: use multiple genes, Filter by median expression, Gene symbol: 203717_at (DPP4), include only patients with high expression of gene 214727_at (BRCA2), start analysis using selected genes, auto select best cutoff, OS, Histology: serous, Stage: 3 + 4, Grade: 3, TP53 mutation: mutated, Debulk: optimal. **h** KM-plotter [23] survival analysis like (**g**) but with the following parameter change: include only patients with low expression of gene 214727_at (BRCA2). Statistical significance of Kaplan-Meier analyses was calculated using Log-rank test or, when groups crossed each other, Gehan-Breslow-Wilcoxon test. Activity assay results represent the mean, error bars are SEM, significant differences were determined by Welch’s ANOVA with Tamhane’s T2 multiple comparisons test.

To investigate the role and possible targetability of DPP4 in human ovarian cancer patients to improve PARPi therapy, human *DPP4* (*hDPP4*) expression was immunohistochemically analysed and semiquantitatively scored in 208 patients with high-grade serous ovarian cancer (HGSOC; Fig. 5a and Table S1). 48% of the patients exhibited *hDPP4* expression of varying degrees in their tumour tissue (Fig. 5b). To investigate whether the hDPP4 staining reflected hDPP4 function, we analysed DPP4 activity in fresh-frozen tumour samples from those patients of the cohort where fresh-frozen material was available (*n* =  33; Fig. 5c). DPP4 activity, categorised to its IHC staining score, formed distinct sample groups.

The samples from staining score 3 displayed a significantly higher DPP4 activity compared with score 0 (mean DPP4 activity, score 0: 114.8 RFU/min, score 1: 499.7 RFU/min, score 2: 411.6 RFU/min, score 3: 1114 RFU/min; Fig. 5c).

Correlation of hDPP4 staining intensity with prognosis showed a significant and clinically meaningful reduction of overall survival in the *hDPP4*-high expressing group compared to the *hDPP4*-low expressing group (median overall survival 16 (score 3) vs. 44 months (score 0-2), *P* < 0.001; Fig. 5d). High *hDPP4* expression remained a prognostic marker for adverse survival also in multivariate analysis (Table 1).

**Table 1:** Multivariate Cox regression analysis of clinical outcome in high-grade serous ovarian cancer patients (FIGO III/IV) with respect to clinical parameters and hDPP4 expression.

Clinical parameters	*n*	PFS	*P*	*n*	OS	*P*
		HR (95% CI)			HR (95% CI)	
FIGO stage			**0.025**			**0.008**
III	138	1		153	1	
IV	41	1.57 (1.06–2.33)		51	1.65 (1.14–2.40)	
Residual tumour mass			**<0.001**			**<0.001**
0 cm	79	1		87	1	
> 0 cm	100	1.89 (1.35–2.65)		117	2.65 (1.84–3.81)	
Nodal status			0.741			0.626
negative	77	1		86	1	
positive	102	1.06 (0.76–1.47)		118	1.09 (0.77–1.54)	
hDPP4 expression			**0.011**			**<0.001**
low	172	1		193	1	
high	7	2.74 (1.27–5.94)		11	4.82 (2.48–9.39)	

Significant values (*P* < 0.05) are indicated in bold.

*CI* confidence interval, *FIGO* International Federation of Gynaecology and Obstetrics. *HR* hazard ratio, *OS* overall survival; *PFS* progression-free survival.

In vivo, lentiviral overexpression of *mDPP4* in ID8-*Trp53*^*(−/−)*^ tumours (Fig. S3F) reduced survival in C57BL/6 mice (Fig. 5e), thus showing that mDPP4 also functionally accelerates tumour growth, although in vitro the clones and their respective control clones exhibited similar sensitivity to olaparib regarding mCCL5 and mCXCL10 induction and a similar proliferation behaviour (Fig. S3G, S3H).

Surprisingly, however, in the ID8-*Trp53*^*(−/−)*^*Brca2*^*(−/−)*^ model, a reverse effect was seen in that *mDPP4* overexpression had a functionally protective role on tumour growth (Fig. 5f). These observations are consistent with publicly available data from the KM plotter when patients are stratified according to their median *hDPP4* expression in *BRCA2*-high expressing and *BRCA2-*low expressing cohorts (Fig. 5g, h), which was the closest parameter to depict *BRCA2* wild-type vs. mutant cohorts. The reasons for this differential effect of *hDPP4* on tumour growth depending on *BRCA2* status need to be further elucidated.

Taken together, *hDPP4* is expressed in a substantial number of high-grade serous ovarian cancers and might thus be a feasible target to improve PARP inhibitor therapy.

## Discussion

The efficacy of PARPi is based on at least two effects: Synthetic lethality-promoted cytotoxicity and STING activation [36]. In this work, we provide additional preclinical indication for the role of STING in PARPi functioning, suggesting to monitor STING activation to find a tailored treatment dose with optimal therapy response. In support of this, we show that chemokine-degrading proteases such as DPP4 confer resistance to PARP inhibition and are attractive targets for enhancing PARPi action even in HRP ovarian carcinomas.

We find that STING activation and cytotoxicity of olaparib have different dynamics in our murine HRD and HRP ovarian cancer cell line models. In vitro, cytotoxicity follows a dose-dependent dynamic, which has been reported for different PARPi in different cell types [29, 37, 38]. The olaparib-induced chemokine induction, however, stagnates at high olaparib concentrations, which may be explained by apoptosis mechanisms overruling chemokine production.

In the immunocompetent *Brca2* knockout model in vivo, we found that reducing the olaparib dose by 50% mediated a significant survival benefit. In contrast, reduction by 90% yielded survival benefits similar to those of the full dose. One striking difference elicited by the different doses was mCXCL10 induction, which was only increased by the lower olaparib doses. This led us to speculate that distinct therapeutic windows might exist for each effect, cytotoxicity, and STING activation. To achieve optimal therapeutic efficacy, the treatment demonstrated its highest effectiveness when employing a precisely calibrated dose of the PARP inhibitor situated within the intersecting region of both therapeutic windows. Following this hypothesis, 50% of the total dose capitalised on both effects. The fact that we did not observe the induction of mCCL5 in our *Brca2* knockout mouse experiments may be explained by epigenetic silencing described in the ID8 model and human HRD ovarian cancer cells [39, 40].

Removing STING-mediated chemokine induction from the equation by treating *Brca2* knockout tumours unresponsive to STING activation resulted in survival benefits that recapitulated the dose-dependent dynamic of cell cytotoxicity by synthetic lethality.

In athymic mice, the survival benefit was reduced to the magnitude of the pure effect of synthetic lethality at best, underlining the importance of an adaptive immune response for PARPi even in the *Brca2* loss-of-function situation. This supports prior reports in preclinical ovarian and breast cancer [7, 9, 11], in some of which the effect of olaparib on tumour burden was completely abolished in *BRCA*-mutated carcinomas by removing the immune component [7], similar to our results in athymic nude mice. The complete loss of the olaparib effect in the *Brca2*^*(−/−)*^ model (in contrast to the *Brca2*^(−/−)*^ model) could be due to tumour-promoting immune cells still being attracted to the *Brca2*^*(−/−)*^ model by intact chemokine secretion in athymic mice lacking mature T cells. The capacity of the same chemokines (including mCCL5 and mCXCL10) attracting tumour-suppressive as well as tumour-promoting immune cells has been reviewed in detail [41].

Since olaparib acts like an indirect STING activator, insights from reports focusing on direct STING agonists may help to decipher why higher doses of olaparib have an adverse effect on survival. STING agonists do not induce tumour cell death in vitro. However, they effectively prompt tumour clearance in various mouse tumour models. This underscores the significance of STING activation in a successful anti-tumour response, emphasising the essential interplay with immune cells [42–44]. Sivick et al. reported that higher doses of the STING agonist ADU S100 led to faster tumour ablation, but lower doses induced most tumour-specific T cells and an immunologic memory with successful rechallenge abrogation [45]. Of note, T cell infiltration upon STING agonist treatment depends on CXCR3 expression [46], and high doses of STING agonists can adversely affect T cells by inducing apoptosis [47, 48]. Taken together, STING-activating PARPi doses appear to have a narrow therapeutic window that is situated below doses that induce maximum cytotoxicity. This is especially important when considering that dose selection of oncological therapeutics is most often determined using the maximum tolerated dose approach [49].

Pivotal clinical studies like SOLO-1 and PAOLA-1 investigating PARP inhibitor treatment of ovarian cancer patients used the same PARPi doses and did not investigate the effect of different dosing, nor did they correlate markers of STING activation with therapy response [3, 50]. As such, the possibility of a distinct therapeutic window of STING activation in response to different PARPi doses has not been assessed. PARPi's emerging immunogenic role could allow for reevaluating the clinically relevant dose. The possibility of reducing PARPi doses is corroborated by the frequently performed PARPi dose reductions in case of side effects, where efficacy is reported not to be impaired [51].

In HRP tumours, STING activation by increasing PARPi doses is not compromised in the same way by cytotoxicity as in HRD tumours because cytotoxicity is significantly weaker in HRP tumours. In line with this, our results indicate that compared with *Brca2*-mutated cells, higher PARPi doses are needed and tolerated to induce chemokines in HRP cells. This is also supported by prior work showing upregulation of type I IFN in a PDX model of *Brca2* WT triple-negative breast cancer at higher olaparib doses than in a *BRCA1*-mutated PDX model [52]. In the *Brca2* wild-type model, we found that the full dose of 50 mg/kgBW olaparib induced ascites chemokines and improved survival, but the effect was modest. To improve on this and to further evaluate the importance of chemokine release for PARPi action, we turned to chemokine-degrading proteases as possible obstacles to successful PARPi therapy. Recently, it was shown that the protease DPP4 reduces the infiltration of lymphocytes by cleaving and inactivating CCL5 and CXCL10 and that the inhibition of DPP4 improves tumour control and synergises with immune checkpoint therapies (ICBs) [53]. Treatment of *mDPP4*-overexpressing ID8-*Brca2* knockout tumours with olaparib did not induce mCXCL10 release, which hints at mCXCL10 degradation by mDPP4. Even though DPP4 has a variety of other substrates, and the causality has yet to be definitively established, our results propose, for the first time, proteolytic chemokine cleavage as a potential PARPi resistance mechanism.

While using the DPP4 inhibitor sitagliptin had no additional benefit to olaparib in the *Brca2*-mutated model (Fig. S2C), it significantly impacted survival in the HRP ovarian cancer model. The survival benefit had the same magnitude as synthetic lethality alone in the *Brca2*-mutated model. In this way, adding an affordable and well-tolerated DPP4 inhibitor might enable PARPi treatment in HRP HGSOC. This could have important implications for HRP HGSOC patients who did not benefit from olaparib in the PAOLA-1 trial [3]. Our results show that about 50% of HGSOC tumours express *hDPP4*, which is probably a conservative estimate, as other research groups show expression rates up to 80% compared to about 17% in benign ovarian tumours [54]. Furthermore, for the first time, we demonstrated DPP4 activity in the tissue of HGSOC patients, which also correlates well with its immunohistochemically detected expression. In our HGSOC patient cohort, high *hDPP4* expression correlated significantly with bad prognosis, and this was recapitulated functionally in our murine *Brca2* wild-type tumour model. Thus, HRP HGSOC patients represent an attractive target population for the use of sitagliptin as an adjuvant for successful PARPi therapy. What remains to be clarified, however, is our observation that *mDPP4* surprisingly showed a protective effect in the *Brca2*-deficient mouse model outside of olaparib therapy, which is supported by our KM-plotter in silico survival analysis. However, the latter should be interpreted with caution, as we used BRCA2 expression as a surrogate for BRCA2 function because the proportion of BRCA2-mutated tumours in publicly available databases was too low to generate reliable statistical power to assess the prognostic significance of DPP4. Nuclear interactions of *mDPP4* with Brca2 or disturbance of the Dpp9-Brca2 axis might account for this observation [55, 56] and will be subject to further research. However, despite its positive prognostic value, DPP4 should be tested as a predictive marker for PARPi resistance, and its inhibition could also further improve PARPi therapy in BRCA-mutated ovarian cancer patients. A prominent example of the therapeutically successful inhibition of a positive prognostic factor is the blockade of the oestrogen receptor in breast cancer.

Taken together, our data fuel the importance of STING activation for the efficacy of PARPi in ovarian cancer. Monitoring STING-induced chemokines in sequential (liquid) biopsies from PARPi-treated HGSOC patients might guide optimal dosing to optimise this effect. Moreover, inhibiting the proteolytic degradation of these chemokines, e.g., by repurposing well-known and well-tolerated drugs such as sitagliptin, could improve PARPi action and PARPi combinations with immune checkpoint inhibitors whose action depends on an inflamed tumour environment.

## Supplementary information

Supplementary Data

## Data Availability

The data generated in this study are available upon request from the corresponding author.
