# Supplementary material for: PARP inhibitor-induced anti-tumour chemokine response is suppressed by dipeptidyl peptidase 4 (DPP4) in ovarian cancer
Source: Br J Cancer. 2025 Jun 27;133(4):582–93. doi: 10.1038/s41416-025-03076-4 (PMC12356936; doi:10.1038/s41416-025-03076-4)
Supplement: Supplementary file 1 — Supplementary Data [file 41416_2025_3076_MOESM1_ESM.docx]

Supplementary data:


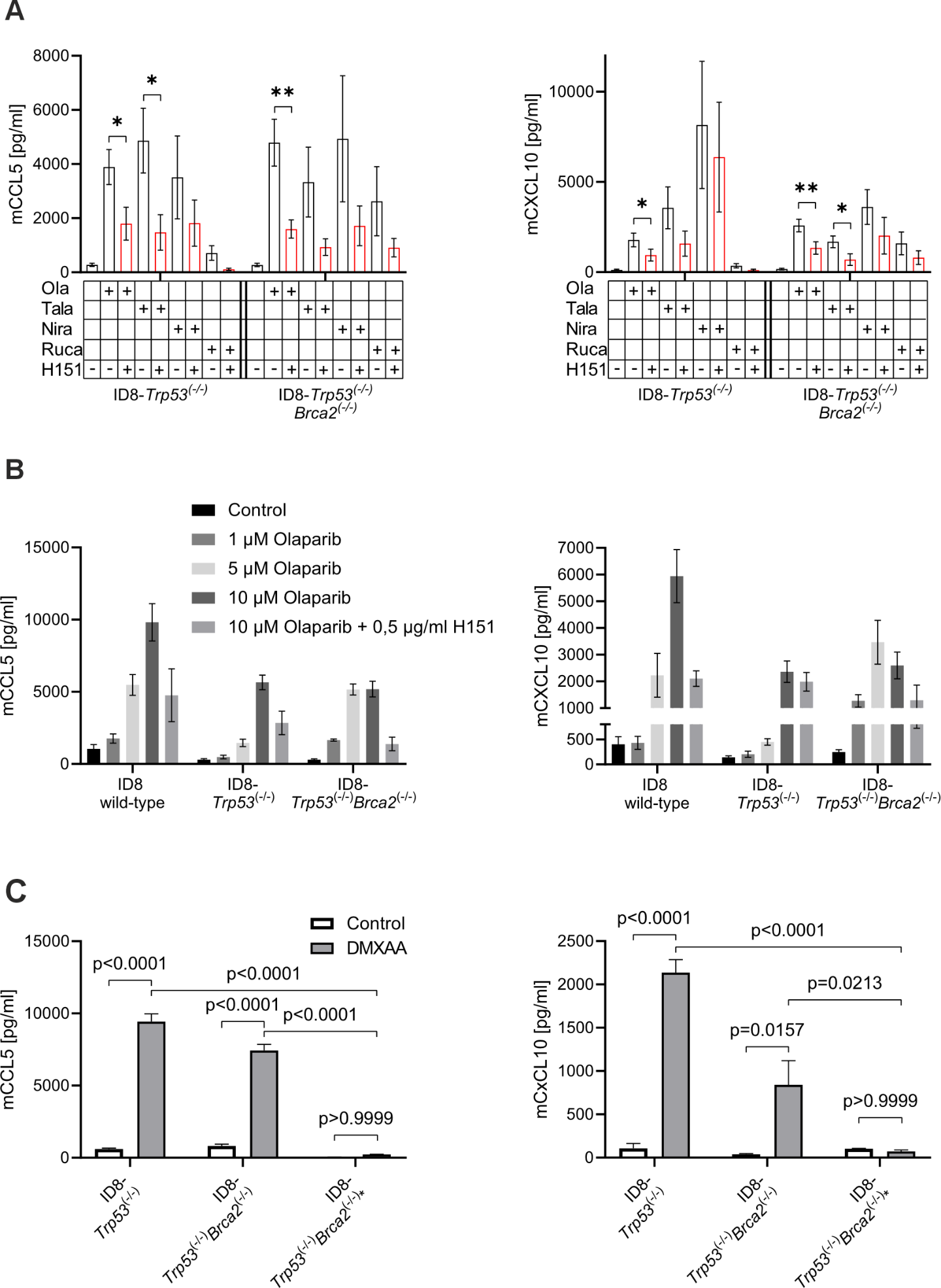


S1: **A** Cell lines ID8-*Trp53*^(-/-)^ and ID8-*Trp53*^(-/-)^*Brca2*^(-/-)^ were stimulated with DMSO as solvent control, 10 µM olaparib, 1 µM talazoparib, 10 µM niraparib or 10 µM rucaparib for 72 h, each with or without the addition of 0.5 µg/mL H151 as indicated. Supernatants were collected and mCCL5 and mCXCL10 concentrations were analysed *via* ELISA, significant differences were determined by two-way ANOVA with Fisher’s LSD test. **B** Cell lines ID8 wild-type, ID8-*Trp53*^(-/-)^ and ID8-*Trp53*^(-/-)^*Brca2*^(-/-)^ were stimulated with DMSO as solvent control, 1 µM olaparib, 5 µM olaparib, 10 µM olaparib or 10 µM olaparib plus 0.5 µg/mL H151 for 72 h. Supernatants were collected, and the concentrations of mCCL5 and mCXCL10 were analyzed *via* ELISA. **C** Cell lines ID8-*Trp53*^(-/-)^, ID8-*Trp53*^(-/-)^*Brca2*^(-/-)^ and ID8-*Trp53*^(-/-)^*Brca2*^(-/-)^* were stimulated with DMSO as solvent control and 0.1 mg/mL DMXAA (HY-10964, MedChemExpress) for 72 h. Supernatants were collected, and the concentrations of mCCL5 and mCXCL10 were analyzed *via* ELISA, significant differences were determined by two-way ANOVA with Sidak's multiple comparisons test. ELISA results represent the mean, means of at least three individual experiments are shown. Error bars are s.e.m, with alpha = 0.05, * p<0.05, ** p<0.01, *** p<0.001.


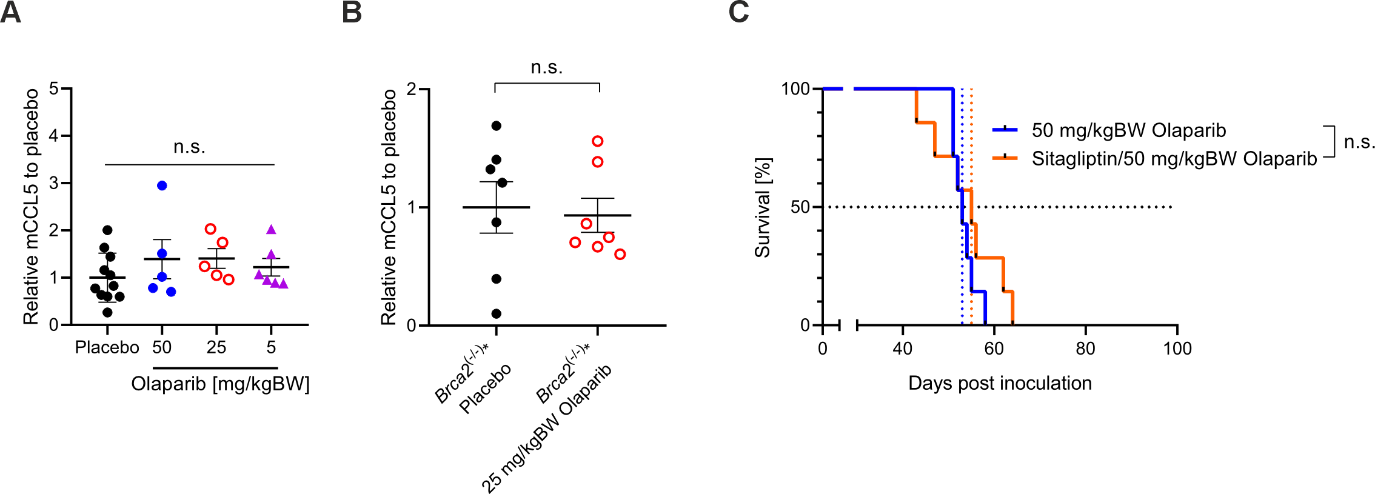


S2: **A** ELISA-quantification of mCCL5 in the ascitic fluid from Fig. 2C, pooled from two individual experiments and normalized to the corresponding mCCL5 mean in the placebo group. **B** ELISA-quantification of mCCL5 in the ascitic fluid from Fig. 2E, pooled from two individual experiments and normalized to the corresponding mCCL5 mean in the placebo group. **C** Kaplan-Meier plot showing survival of C57BL/6 mice intraperitoneally inoculated with syngeneic ID8-*Trp53*^(-/-)^*Brca2*^(-/-)^ and treated with 50 mg/kgBW olaparib (n=7) and 50 mg/kgBW sitagliptin (n=7) as indicated. The statistical significance of Kaplan-Meier analyses was calculated using the Gehan-Breslow-Wilcoxon test. ELISA results represent the mean, error bars are s.e.m, significant differences were determined by two-way ANOVA with Tukey’s multiple comparisons test in S2A and unpaired t-test in S2B, with alpha = 0.05, * p<0.05, ** p<0.01, *** p<0.001.


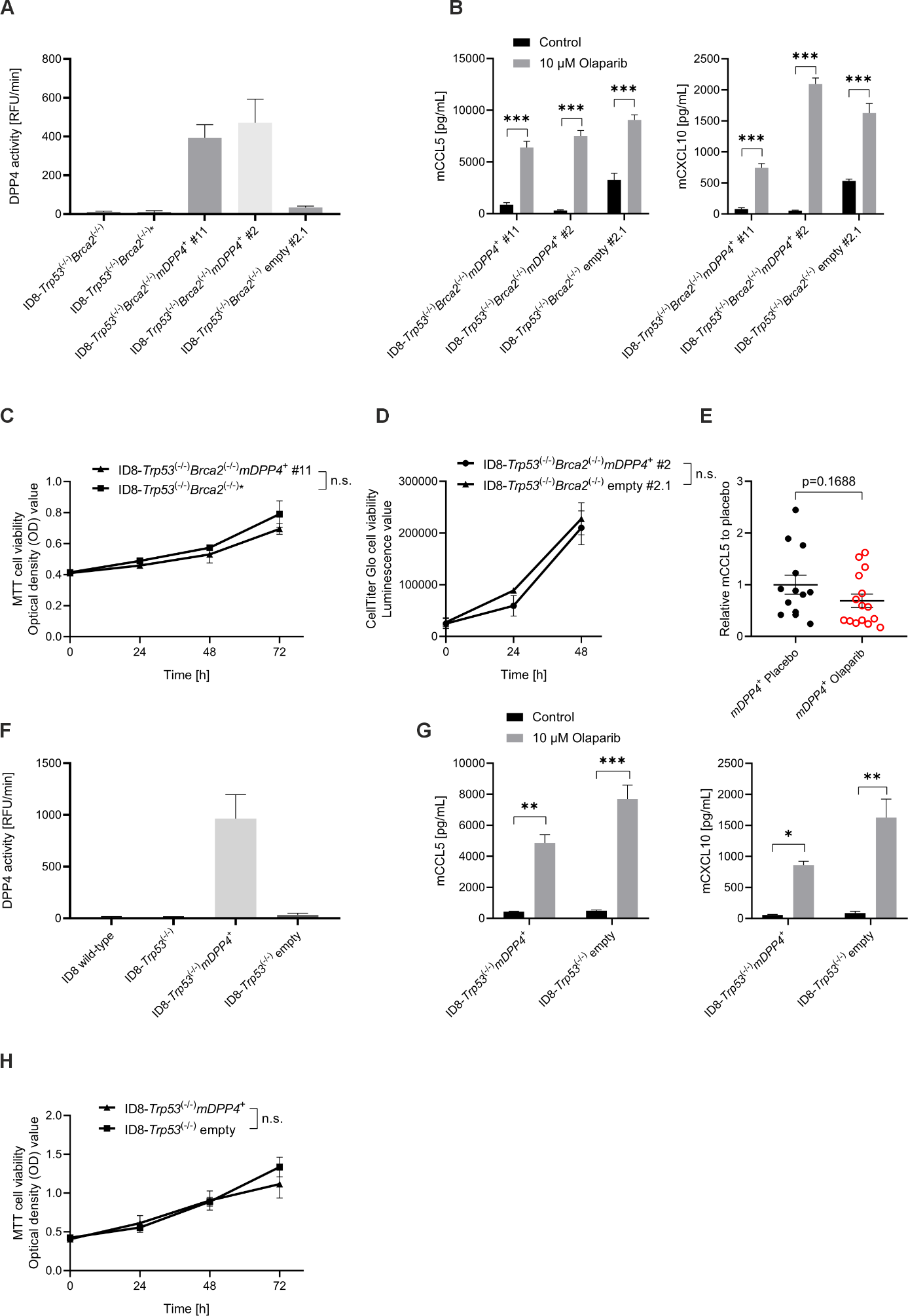


S3: **A** Activity assay displaying DPP4 activity in cell lysates of ID8-*Trp53*^(-/-)^*Brca2*^(-/-)^ parental cells, ID8-*Trp53*^(-/-)^*Brca2*^(-/-)^* and *mDPP4*-overexpressing (#11, #2) or empty vector control cells (#2.1). Cell line #11 has been used in the animal experiment shown in Fig. 3, and cell lines #2 and #2.1 have been used in the animal experiment shown in Fig. 5F. **B** The indicated cell lines were stimulated with DMSO or 10 µM olaparib for 72 h. Supernatants were collected, and mCCL5 and mCXCL10 concentrations were analyzed *via* ELISA, significant differences were determined by two-way ANOVA with Fisher’s LSD test. **C** MTT assay showing no significant differences between ID8-*Trp53*^(-/-)^*Brca2*^(-/-)^*mDPP4*^+^ #11 and ID8-*Trp53*^(-/-)^*Brca2*^(-/-)^*, significant differences were determined using two-way ANOVA with Sidak’s multiple comparisons test. **D** CellTiterGlo proliferation assay showing no significant differences between ID8-*Trp53*^(-/-)^*Brca2*^(-/-)^*mDPP4*^+^ #2 and ID8-*Trp53*^(-/-)^*Brca2*^(-/-)^ empty #2.1, significant differences were determined using two-way ANOVA with Sidak’s multiple comparisons test. **E** ELISA-quantification of mCCL5 in the ascitic fluid from Fig. 3A, pooled from two individual experiments and normalized to the corresponding mCCL5 mean in the placebo group, significant differences were determined using unpaired t-test. **F** Activity assay displaying DPP4 activity in ID8-*Trp53*^(-/-)^ parental cell lysates and *mDPP4*-overexpressing or empty vector control cell lysates. The *mDPP4*-overexpressing cell line and its empty vector control have been used in the animal experiment shown in Fig. 5E. **G** The indicated cell lines were stimulated with DMSO or 10 µM olaparib for 72 h. Supernatants were collected, and mCCL5 and mCXCL10 concentrations were analyzed *via* ELISA. Significant differences were determined by two-way ANOVA with Fisher’s LSD test. **H** MTT assay showing no significant differences between ID8-*Trp53*^(-/-)^*mDPP4*^+^ and ID8-*Trp53*^(-/-)^, significant differences were determined using two-way ANOVA with Sidak’s multiple comparisons test. ELISA, proliferation assay, and activity assay results represent the mean, error bars are s.e.m, significant differences were determined as described for each plot, with alpha = 0.05, without assuming consistent SD, * p<0.05, ** p<0.01, *** p<0.001.

Supplementary methods:

**CellTiter Glo proliferation assay.** 2000 cells per well were seeded into one 96 well plate (83.3924, Sarstedt) in 100 µL of fully supplemented culture media. After 90 min, 24 h, and 48 h, 100 µl CellTiter Glo 2.0 reagent was added to the corresponding wells, lysed for 2 min on an orbital shaker, and incubated at room temperature for 10 min. Luminescence was measured directly afterwards using the default luminescence program of the Victor X2 multilabel reader (PerkinElmer), capturing photons for 1 s per well.

**DMXAA-mediated stimulation of mCCL5 and mCXCL10.** 3 x 104 cells were seeded into 12 well plates in fully supplemented culture media. The next day, cells were stimulated with 0.1 mg/mL DMXAA (HY-10964, MedChemExpress) in DMEM supplemented with 5% (v/v) FBS. After 72 h of incubation in the cell incubator, cell supernatant was collected for ELISA analysis.

**Table S1.** Patient demographics and clinicopathologic characteristics of the high-grade serous ovarian cancer patient cohort (n = 208).

| Clinical parameters | |  |  | n | range or % |
| --- | --- | --- | --- | --- | --- |
|  |  |  |  |  |  |
| Median age at diagnosis (years) (range) | | | |  | 69 (33-88) |
| ≤ 60 at initial diagnosis | |  |  | 77 | (37.0) |
| > 60 at initial diagnosis | |  |  | 131 | (63.0) |
|  |  |  |  |  |  |
| Median follow up time PFS (months) (range) | | | | 17 | (1-74) |
| Median follow up time OS (months) (range) | | | | 42 | (1-269) |
|  |  |  |  |  |  |
| FIGO stage |  |  |  |  |  |
| III |  |  |  | 159 | (75.0) |
| IV |  |  |  | 52 | (25.0) |
|  |  |  |  |  |  |
| Postsurgical residual tumour mass | | |  |  |  |
| 0 cm |  |  |  | 87 | (41.8) |
| > 0 cm |  |  |  | 121 | (58.2) |
|  |  |  |  |  |  |
| Nodal status | |  |  |  |  |
| Negative (pN0) | |  |  | 86 | (41.3) |
| Positive (pN1) | |  |  | 118 | (57.8) |
| No data available | |  |  | 4 | (1.9) |
|  | |  |  |  |  |

**FIGO**, International Federation of Gynecology and Obstetrics; **OS**, overall survival; **PFS**, progression-free survival.
